# Efficacy of neuromuscular electrical stimulation for thoracic and abdominal surgery: A systematic review and meta-analysis

**DOI:** 10.1371/journal.pone.0294965

**Published:** 2023-11-30

**Authors:** Yuki Nakashima, Daisuke Iwaki, Yuki Kataoka, Takashi Ariie, Shunsuke Taito, Yuichi Nishikawa, Naoki Mio, Yukio Mikami

**Affiliations:** 1 Division of Rehabilitation, Department of Clinical Practice and Support, Hiroshima University Hospital, Hiroshima, Japan; 2 Scientific Research WorkS Peer Support Group (SRWS-PSG), Osaka, Japan; 3 Department of Neuromechanics, Graduate School of Biomedical and Health Sciences, Hiroshima University, Hiroshima, Japan; 4 Department of Internal Medicine, Kyoto Min-iren Asukai Hospital, Kyoto, Japan; 5 Section of Clinical Epidemiology, Department of Community Medicine, Kyoto University Graduate School of Medicine, Kyoto, Japan; 6 Department of Healthcare Epidemiology, Kyoto University Graduate School of Medicine / Public Health, Kyoto, Japan; 7 Department of Physical Therapy, School of Health Sciences at Fukuoka, International University of Health and Welfare, Fukuoka, Japan; 8 Faculty of Frontier Engineering, Institute of Science & Engineering, Kanazawa University, Kanazawa, Japan; 9 Department of Rehabilitation Medicine, Hiroshima University Hospital, Hiroshima, Japan; University of Brasilia, BRAZIL

## Abstract

This systematic review and meta-analysis examined the efficacy of neuromuscular electrical stimulation (NMES) on lower limb muscle strength and health-related quality of life (HR-QOL) after thoracic and abdominal surgery. We searched the Cochrane Central Register of Controlled Trials, MEDLINE via PubMed, Excerpta Medica Database via Elsevier, Physiotherapy Evidence Database, Cumulative Index to Nursing and Allied Health Literature, World Health Organization International Clinical Trials Registry Platform via their dedicated search portal, and ClinicalTrials.gov on November 2021 and updated in April 2023 to identify randomized controlled trials that examined the effects of NMES after thoracic and abdominal surgery. The primary outcomes were lower limb muscle strength, HR-QOL, and adverse events. We used the Cochrane Risk of Bias Tool and the Grading of Recommendations, Assessment, Development, and Evaluation approach to assess the certainty of evidence. A total of 18 randomized control trials involving 915 participants, including 10 on cardiovascular surgery, two on pulmonary surgery, five on digestive system surgery, and one on other surgery, were included. NMES slightly increased lower limb muscle strength and adverse events in cardiovascular surgery. Adverse events (hypotension, pain, and muscle discomfort) occurred in seven patients. HR-QOL was measured in two studies on cardiovascular surgery, but these were not pooled due to concept heterogeneity. Overall, NMES slightly increases lower limb muscle strength after cardiovascular surgery without serious adverse events. However, higher-quality randomized control trials in thoracic and abdominal surgeries are needed.

## Introduction

After major surgery, patients often experience a decline in the ability to carry out activities of daily living (ADL) and walking speed. A prospective cohort study reported that 9% of patients experience disabilities in ADL and 39% of patients had decreased walking speed 6 months after abdominal surgery [1]. Physical functions such as muscle strength and walking speed have been reported to decrease after thoracic and abdominal surgery [2–5]; therefore, preventing a decline in physical function is important in the postoperative period. Early mobilization is essential to prevent postoperative complications and loss of physical function; however, pain and fatigue often impede recovery [6], with 58% of patients failing to achieve this goal [7]. A previous systematic review reported little impact of early mobilization protocols on performance-based outcomes [8]. Thus, maintaining muscle strength and walking ability remains challenging.

Neuromuscular electrical stimulation (NMES) improves muscle strength by delivering intermittent electrical stimulation, through electrodes attached to the skin, to the skeletal muscles which cause muscle contractions [9]. NMES has been used as an alternative to exercise in patients with decreased physical activity, such as in post stroke patients [10]. Furthermore, a systematic review reported that NMES effectively increased lower limb muscle strength after orthopedic surgery [11, 12].

A systematic review regarding the efficacy of NMES on lower limb muscle strength was reported after thoracic and cardiac surgery [13]. However, this systematic review meta-analyzed randomized controlled trials (RCTs) and a non-RCT, which may have introduced false estimates of effect sizes. Additionally, to date, no systematic reviews of NMES have been conducted for other thoracic surgeries, such as pulmonary and abdominal surgeries. Therefore, we examined whether NMES improved outcomes, such as physical function (e.g., lower extremity muscle strength) and health-related quality of life (HR-QOL) after thoracic and abdominal surgery.

## Methods

We conducted a systematic review and meta-analysis. We followed the Preferred Reporting Items for Systematic Review and Meta-analysis 2020 (PRISMA-2020) guidelines (S1 Table) [14]. We registered our research protocols using the Open Science Framework (https://osf.io/3rdvf/).

### Inclusion criteria of the articles for the review

#### Type of studies

We included RCTs that assessed individual randomization, crossover randomization, and cluster randomization. Language and country restrictions were not applied. We also included all papers, including letters, conference abstracts, and published and unpublished articles. We did not exclude studies based on observation period or publication year.

### Study participants

#### Inclusion criteria

The target population included patients aged ≥18 years who had undergone thoracic and abdominal surgery. In this study, thoracic surgery included cardiac surgery (e.g., coronary artery bypass, aortic valve surgery, aortic surgery, and heart transplantation), pulmonary surgery (e.g., partial or total lung lobectomy and lung transplantation), and esophagectomy. In addition, patients who underwent open or minimally invasive abdominal surgery were included. There were no restrictions on the diseases that could be treated. For instance, patients with benign or malignant tumors or organ transplants were anticipated.

We classified thoracic and abdominal surgeries into cardiovascular, pulmonary, digestive system, and other surgeries.

#### Exclusion criteria

We excluded endovascular procedures, such as transcatheter aortic valve implantation. In addition, studies that included mixed populations, where a percentage of participants had only endovascular treatment or were children (<18 years), were excluded unless the results of patients who underwent surgery were presented separately or there were only a few (<5%) endovascular treatments or patients aged <18 years.

## Intervention

### NMES definition

NMES is a technique for boosting muscle strength by attaching electrodes to the skin and delivering a series of intermittent electrical stimulations to the skeletal muscles. This causes muscle contractions by activating the nerves to the motor branches of the muscles [9].

To examine the effect of early postoperative NMES intervention, we included studies in which NMES intervention was initiated from the day of surgery to postoperative day 7. Additionally, we included studies that examined NMES (one or more times) as a standalone intervention or along with the usual rehabilitation. Although the quadriceps is often the primary stimulation site, studies using NMES on lower limb muscle groups such as hamstrings, gastrocnemius, and buttocks, and interventions using multiple stimulation sites, were all included. Furthermore, we excluded studies on NMES interventions targeting only the upper limb or pelvic floor muscles and for providing electrical stimulation for pain relief.

We expected the programs to differ in stimulus frequency (Hz), pulse type, pulse duration (μs), duty cycle (%), session duration (min), frequency (sessions/week), and overall program duration (weeks). Nevertheless, there were no restrictions based on these parameters. Therefore, it was acceptable for the intervention group to undergo normal rehabilitation and care.

### Control

The control group included no treatment, placebo, sham interventions (e.g., no output of the stimulator or stimulation parameters below the level needed to promote muscle contraction), usual rehabilitation, or routine care.

### Type of outcomes

The primary outcomes were lower limb muscle strength, HR-QOL, and adverse events (defined by trialists). Secondary outcomes included walking ability, activities of daily living (ADL), length of stay in the intensive care unit (ICU), and length of hospital stay. For lower limb muscular strength, HR-QOL, walking ability, and ADL, we defined the outcome time points within 1 month of surgery and during the intervention and follow-up period for adverse events.

### Search strategy and selection of studies

We searched the Cochrane Central Register of Controlled Trials, MEDLINE via PubMed, Excerpta Medica Database via Elsevier, Physiotherapy Evidence Database, Cumulative Index to Nursing and Allied Health Literature, World Health Organization International Clinical Trials Registry Platform via their dedicated search portal, and ClinicalTrials.gov in November 2021 and updated in April 2023.

We used suitable search terms, including surgery, thoracic surgery, colorectal surgery, and organ transplantation, to search for population and neuromuscular electrical stimulation for intervention (S1 Appendix). International guidelines, eligible studies, and articles citing eligible studies were also examined [15–23]. For unpublished or additional data, we contacted the authors of the original studies. To determine whether each study returned by the search met the inclusion criteria, two reviewers (YN and DI) independently examined the title and abstract of each study. In addition, they performed a full-text review to assess the inclusion eligibility of every candidate study. Disagreements were resolved by discussion between the two reviewers and occasionally by a third reviewer (TA) arbitrate.

### Assessment of risk of bias in included studies

YN and DI independently evaluated the risk of bias using the Risk of Bias 2 [24]. Disagreements between the two reviewers were resolved through discussion, and if this failed, TA acted as an arbiter if necessary.

### Measures of treatment effects

We pooled the mean differences (MD) and the 95% confidence interval (CI) for the following continuous variables: walking ability in digestive system surgery, ADL, length of stay in ICU, and hospital stay duration. We pooled the effect estimates using standard MD (SMD) for lower limb muscle strength, HR-QOL, and walking ability in cardiovascular surgical procedures. Finally, we pooled the relative risk ratios and 95% CIs for the binary variable: adverse events.

### Unit of analysis issues

For continuous outcomes, we used the data according to the following hierarchy:

- First-period data
- MD between the intervention and control periods and the standard deviation (SD)
- If the SD above was unavailable, 95% CI, t-statistic, or p-value for the t-test were used
- If any above statistics were unavailable, we performed approximate analyses to impute the SD of the MD between the intervention and control periods according to the Cochrane Handbook Chapter 23.2.7 [25].

### Handling of missing data

We asked the original authors about missing data. For all dichotomous data, we extracted the data on an intention-to-treat basis whenever feasible. Based on Cochrane Handbook’s recommendations, we did not impute missing data for continuous data [26]. We conducted a meta-analysis using the information from the original research. If the authors could not provide us with these numbers when contacted, the SD was determined using the CI and t-value method described in the Cochrane Handbook [26], which is a validated method [27].

### Assessment of heterogeneity

By visually inspecting the forest plots and calculating the I^2^ statistic, we assessed the statistical heterogeneity (I^2^ values of 0% to 40% may not be significant, 30% to 60%: moderate heterogeneity, 50% to 90%: substantial heterogeneity, and 75% to 100%: considerable heterogeneity) [26].

### Assessment of reporting bias

We searched clinical trial registration systems (ICTRP and ClinicalTrials.gov) and conducted an extensive literature search for unpublished trials. We compared the outcomes specified in the trial protocols with those detailed in the publications to evaluate the bias in outcome reporting. Visual inspection of funnel plots was used to evaluate potential publication bias. We did not perform an Egger test due to the sample size.

### Meta-analysis

We performed a meta-analysis using the Review Manager software (RevMan 5.4.2). We used a random-effects model.

### Subgroup analysis

We performed subgroup analysis according to the stimulation site (one muscle vs. multiple muscles).

### Difference between protocol and review

We performed a meta-analysis for adverse events because there was little or no heterogeneity of adverse events. Moreover, due to insufficient data, we could not perform planned subgroup analyses for the following variables: age (65 vs. >65 years) and treatment frequency (five vs. five times/week). In addition, owing to insufficient data, we were also unable to conduct the intended sensitivity analyses for the primary outcomes: exclusion of studies: (i) using imputed statistics; (ii) with a high risk of bias of overall risk of bias; and (iii) that randomly assigned NMES to one limb of a person and the other limb received control.

### Summary of findings table

Two reviewers (YN, TA) evaluated the certainty of evidence based on the Grading of Recommendations Assessment, Development, and Evaluation (GRADE) approach [28]. We resolved disagreements by discussing them between the two reviewers and occasionally by having a third reviewer (YK) arbitrate. The participants were classified into four categories: cardiovascular surgery, pulmonary surgery, digestive system surgery, and other surgeries. We summarized the findings for the following outcomes based on the Cochrane Handbook [26]. We used the GRADE informative statement to report each outcome [29].

1. Lower limb muscle strength
2. HR-QOL
3. Measured as the number of adverse events
4. Walking ability
5. ADL
6. Length of stay in the ICU
7. Length of stay at the hospital

## Results

**Fig 1:**
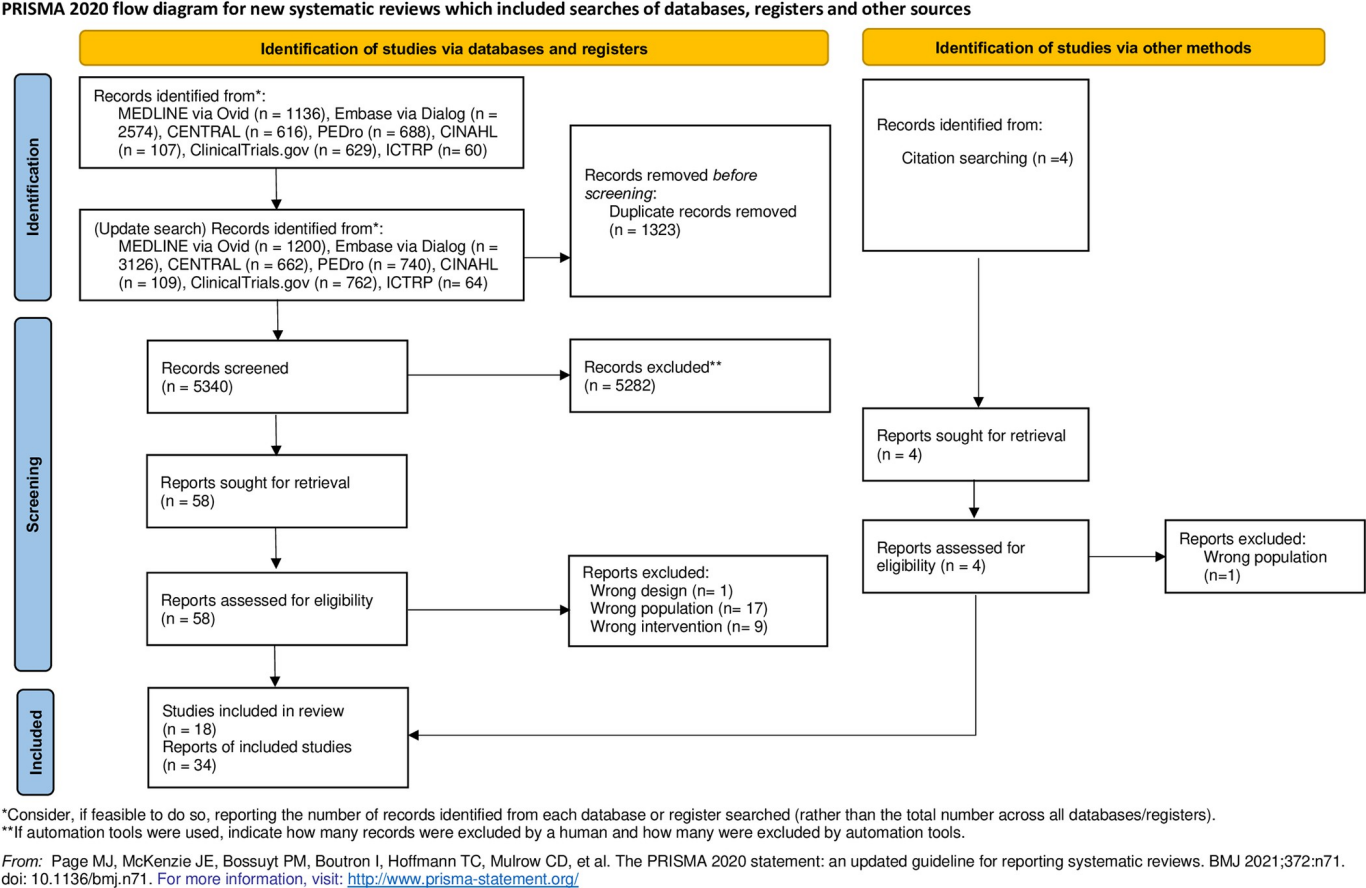
PRISMA flow chart.

In addition to the November 2021 search, an update search was conducted in April 2023. Subsequently, duplicates were removed, resulting in a total of 5340 screenings. After screening, we excluded 17 studies due to incorrect population, nine for incorrect intervention, and one for incorrect design (S2 Table). In the qualitative synthesis, we identified 18 RCTs [30–47] that met all the eligibility criteria (Fig 1 and Table 1). The 18 RCTs included 915 patients who underwent thoracic and abdominal surgeries and were postoperatively treated with NMES. Out of the 18 RCTs, four [33, 36, 41, 44] were only protocol registrations and did not have outcome data.

**Table 1:** Characteristics of included studies: (a) cardiovascular surgery (b) pulmonary surgery (c) digestive system surgery (d) other surgery.

**(a)**						
Author Year	Number of Participants	Types of surgery	Intervention	Stimulation site	Controls	Outcomes
Fischer [30] 2016	54	AVR, CABG, HT, MVR, MVr, TVr, Bentall procedure	Initiation of NMES: postoperative day (POD) 1, frequency of NMES: 7 (day/week), program duration: 2 (weeks), stimulation frequency: 66 (Hz), pulse type: biphasic rectangular pulses, pulse duration: 400 (μs), duty cycle: 3.5 s on and 4.5 s off, session duration: 30×2 (minutes), intervention intensity: Highest tolerable intensity just below the pain threshold	Quadriceps muscle of both thighs	Sham interventions	Muscle layer thickness MRC HGS FIM score Timed Up and Go test SF-12 The average mobility level proposed by Brown Patient satisfaction
Rengo [31] 2021	37	CABG	Initiation of NMES: POD 4.6 ± 0.2, frequency of NMES: 4 (day/week), program duration: 4 (weeks), stimulation frequency: 25 (Hz), pulse type: biphasic pulses, pulse duration: 400 (μs), duty cycle: 25% (10 s on, 30 s off), session duration: 45 (min), intervention intensity: To achieve the maximal tetanic contractions possible within the patient’s pain tolerance	Quadriceps both legs	No intervention	SPPB SF-36 6MWD Physical activity
Kitamura [32] 2019	119	CABG, Valvular, Thoracic aorta	Initiation of NMES: 3 days prior to surgery, POD1, frequency of NMES: Three days before surgery and daily from POD 1 to POD5, program duration: 8 days, stimulation frequency: NA, pulse type: A direct electrical current with a symmetric and biphasic square, pulse duration: NA, duty cycle: With 30-s intervals, ten pulse trains (10 s), session duration: 30 (min), intervention intensity: set at 10% and 20% of the maximal voluntary contraction possible	Bilateral quadriceps femoris and triceps surae muscles	Usual rehabilitation and care	KEIS 3-Methylhistidine/Creatinine Usual walking speed(10m) HGS
Guizilini [33] 2016	protocol	CABG	Initiation of NMES: POD1, frequency of NMES: 5 (day/week), program duration: 1 (weeks), stimulation frequency: 50 (Hz), pulse type: NA, pulse duration: 400 (μs), duty cycle: 10 s on and 20 s off, session duration: NA, intervention intensity: Intensity as tolerated by the patient	Quadriceps and gastrocnemius muscles	Walking exercise or Stationary cycling exercise	6MWD TNF-α Interleukins
Cerqueira [34] 2018	59	AVR, AVr, MVR, MVr	Initiation of NMES: immediately after admission to the postoperative ICU, frequency of NMES: 5 (day/week), program duration: 1 (weeks), stimulation frequency: 50 (Hz), pulse type: NA, pulse duration: 400 (μs), duty cycle: 3 s on and 9 s off, session duration: 60×2 (min), intervention intensity: Adjusted until visible muscle contraction occurred	Bilateral quadriceps and gastrocnemius muscle bellies,	Usual rehabilitation and care	6MWD Gait speed MRC FIM Nottingham Health Profile
Sumin [35] 2020	37	CABG, AVR, MVR, Aortic dissection, HT, Multivalve operations, Bental surgery	Initiation of NMES: POD3, frequency of NMES: Postoperative day 3 to discharge from hospital (12 sessions or more), stimulation frequency: 45 (Hz), pulse type: rectangular pulses, pulse duration: NA, duty cycle: 12 s on and 5 s off, session duration: 90 (min), intervention intensity: until a visible or palpable muscle contraction	Bilateral quadriceps femoris muscle	Usual rehabilitation and care	Knee extensors strength HGS Knee flexor strength Cross-sectional area of the Quadriceps femoris 6MWT
Kiryu [36] 2020	protocol	open heart surgery	Initiation of NMES: operation day, frequency of NMES: 7 (day/week), program duration: 1 (weeks), stimulation frequency: NA (Hz), pulse type: NA, pulse duration: NA (μs), duty cycle: NA, session duration: 30 (min), intervention intensity: NA	Bilateral quadriceps, hamstrings, triceps surae, tibial anterior muscles	Usual rehabilitation and care plus sham	Length of days to walk 100 m Postoperative length of hospital stay The rate of discharge at home Muscle quantity HGS Knee extension strength Gait speed SPPB One leg standing time 6MWD
Takino [37] 2023	180	CABG, Valvular, Thoracic aorta, Other	Initiation of NMES: POD1, frequency of NMES: 7 (day/week), program duration: 1 (weeks), stimulation frequency: 20–200 (Hz), pulse type: symmetric and biphasic square pulses, pulse duration: NA (μs), duty cycle: 0.4 s on and 0.6 s off, session duration: 60 (min), intervention intensity: trigger significant muscular contraction with the highest tolerance level.	Vastus lateralis, vastus medialis, and triceps surae bilaterally	Usual rehabilitation and care plus sham	KEIS 10 m-walk test usual walking speed maximum walking speed grip strength
Cerqueira [38] 2022	45	CABG, MVR, AVR, MVR+ AVr	Initiation of NMES: immediate postoperative period, Frequency of NMES: 5 (day/week), program duration: 1 (weeks), stimulation frequency: 50 (Hz), pulse type: NA, pulse duration: 400 (μs), duty cycle: 3 s on and 9 s off, session duration: 60×2 (min), intervention intensity: until a palpable muscle contraction.	Rectus femoris, gastrocnemius muscles bilaterally	Physiotherapy treatment	6MWD Gait speed Lactate level MRC HGS KEIS Electromyography FIM
ÖZÜBERK [39] 2022	40	CABG	Initiation of NMES: POD2, frequency of NMES: 5 (day/week), program duration: 1 (weeks), stimulation frequency: 25 (Hz), pulse type: NA, pulse duration: NA, duty cycle: 5 s on and 5 s off, session duration: 30 (min), intervention intensity: NA	Bilateral quadriceps and Gastrosoleus muscle	Cardiopulmonary rehabilitation	Myocardial Tissue Doppler 2 Minutes Walk Test 30 Seconds Sit To Stand Up Test Chest Wall Measurements
(b)						
Author Year	Number of Participants	Participants	Intervention	Stimulation site	Controls	Outcomes
Timofte [40] 2021	6	Lung Transplantation	Initiation of NMES: 72 hours post-transplantation, Frequency of NMES: 7 (day/week), program duration: NA, stimulation frequency: NA(Hz), pulse type: NA, pulse duration: NA(μs), duty cycle: NA, session duration: 10–30×1–2 (mins), intervention intensity: NA	Bilateral quadriceps and dorsiflexors	Usual rehabilitation and care	Change in lower extremity skeletal muscle area Average time of intubation
Zaragoza-García [41] 2022	protocol	Lung Transplantation	Initiation of NMES: 48 hours post-transplantation, Frequency of NMES: 7 (day/week), program duration: until discharge, stimulation frequency: NA (Hz), pulse type: NA, pulse duration: NA (μs), duty cycle: NA, session duration: 30×2 (mins), intervention intensity: NA	Lower limb	No Intervention	Variation of muscle mass in the quadriceps IMS Leg strength according to Chair and Stand test
(c)						
Author Year	Number of Participants	Types of surgery	Intervention	Stimulation site	Controls	Outcomes
André [42] 2021	39	Bariatric surgery	Initiation of NMES: within 1 week after surgery, Frequency of NMES: 5 (day/week), program duration: 6 (weeks), stimulation frequency: Endurance 85 Hz, Strength 30 Hz, pulse type: rectangular pulses, pulse duration: 350 (μs), duty cycle: Endurance 6 s on and 4 s off, Strength 4 s on and 10 s off, session duration: 20–30 (min), intervention intensity: current sensitivity, respecting visual and effective contraction, without pain or discomfort	Both arms, thighs, and gluteal region	Exercise plus sham	Cardiopulmonary Exercise Testing 6MWD Isokinetic, isometric, and endurance peripheral dominant knee muscle
Hanada [43] 2019	45	Living donor liver transplant	Initiation of NMES: POD1, frequency of NMES: 5 (day/week), program duration: 4 (weeks), stimulation frequency: 45 (Hz), pulse type: biphasic, symmetrical impulses, pulse duration: 400 (μs), duty cycle: 12 s on and 6 s off, session duration: 30 (min), intervention intensity: increased to elicit visible muscle contractions and to the maximum level tolerated by the patients (40–80 mA)	Bilateral quadriceps muscles	Usual rehabilitation and care plus sham	Quadriceps strength Handgrip force Quadriceps muscle thickness SPPB 6MWD Barthel Index
Pring [44] 2021	Protocol	Locally advanced rectal cancer	Initiation of NMES: 2 weeks before surgery, frequency of NMES: 7 (day/week), program duration: 2 weeks before surgery to 8 weeks after surgery, stimulation frequency: 40 (Hz), pulse type: pulse waveform (symmetrical biphasic squared), pulse duration: 400 (μs), duty cycle: NA, session duration: 60–90 (min), intervention intensity: The amplitude (device output 0–120 mA, tested across 1000 Ω) will be set to elicit a visible and comfortable muscle contraction; patients will be encouraged to increase the amplitude as tolerated subsequently	Quadricep muscles and paraspinal muscles	Placebo NMES and standard care	The difference in mean muscle attenuation Systemic inflammation Cellular immune response Postoperative complications Length of hospital stay Disease-free survival Overall survival EQ-5D-5L EORTC QLQ–CR29
Strasser [45] 2009	18 (split body randomized control trial)	Hemicolectomy, Pancreatectomy, Hemihepatectomy, Dissection of paraaortal lymph nodes, Aortofemoral bifurcation bypass	Initiation of NMES: POD1, frequency of NMES: 4 (day/week), program duration: 1 (weeks), stimulation frequency: 50 (Hz), pulse type: NA, pulse duration: 250 (μs), duty cycle: 8 s on, 4 s off, session duration: 30 (min), intervention intensity: Adjusted to ensure maximum tolerable muscle contraction	Quadriceps femoris	Control leg (Placebo NMES)	mRNA level of IGF-1Ea mRNA level of MGF Total RNA content Total protein content, Ubiquitin-conjugated proteins Proteasome activity.
Hardy [46] 2022	15 (split body randomized control trial)	Open major colonic resection	Initiation of NMES: POD1, frequency of NMES: 4 (day/week), program duration: 1 (weeks), stimulation frequency: 30 (Hz), pulse type: NA, pulse duration: NA, duty cycle: 1 s on and 1 s off, session duration: 15×2 (min), intervention intensity: at the minimum level necessary to cause uncontrolled movement of the knee joint and observable muscle contraction.	Proximally and distally over the lateral quadriceps	Control leg	Vastus Lateralis cross-sectional area knee extensor strength
(d)						
Author Year	Number of Participants	Participants	Intervention	Stimulation site	Controls	Outcomes
Xie [47] 2020	221	Kidney transplant SPK transplant	Initiation of NMES: POD1, frequency of NMES: 6 (day/week), program duration: 1 (weeks), stimulation frequency: NA (Hz), pulse type: NA, pulse duration: NA, duty cycle: NA, session duration: NA, intervention intensity: NA	Common peroneal nerve	Intermittent pneumatic compression plus thrombo-embolic-deterrent.	Calf circumference Urine output Length of stay Occurrence of delayed graft function Number of dialysis sessions postoperatively Renal blood flow Mobility (Steps)

AVR, aortic valve replacement; CABG, coronary artery bypass grafting; HT, heart transplantation; MVR, mitral valve replacement; MVr, mitral valve reconstruction; TVr, tricuspid valve reconstruction; AVr, aortic valve reconstruction; NMES, neuromuscular electrical stimulation; NA, Not Applicable; MRC, Medical Research Council; HGS, Hand grip strength FIM, Functional Independence Measure; SPPB, Short Physical Performance Battery; 6 MWD, six minute walking distance; KEIS, knee extensor isometric muscle strength; TNF, tumor necrosis factor; ICU, intensive care unit; IMS, mobility assessment according to ICU-Mobility scale; EQ-5D-5L, EuroQol 5 dimensions 5-level; EORTC QLQ–CR29, European Organization for Research and Treatment of Cancer Quality of Life Questionnaire-Colorectal Cancer 29; RNA, Ribonucleic acid; IGF, insulin-like growth factor; MGF, mechano-growth factor; SPK, simultaneous pancreas-kidney

Overall, there were 10 studies [30–39] in cardiovascular surgery, two [40, 41] in pulmonary surgery, five [42–46] in digestive system surgery, and one [47] in other surgeries. Cardiovascular surgery studies included aortic valve replacement, coronary artery bypass grafting, heart transplantation, mitral valve replacement, mitral valve reconstruction, or tricuspid valve reconstruction. Pulmonary surgery included lung transplantation. Digestive system surgery included bariatric surgery, living donor liver transplant, locally advanced rectal cancer, hemicolectomy, pancreatectomy, hemihepatectomy, dissection of para-aortal lymph nodes, aortofemoral bifurcation bypass, and open major colonic resection. Other surgical included kidney transplants and simultaneous pancreas-kidney transplants.

The length of the intervention spanned 5 days to 4 weeks, and the frequency of the intervention ranged from three times per week to every day. Seven studies involved stimulation of one muscle [30, 31, 35, 39, 43, 45, 46], nine involved stimulations of multiple muscles [32–34, 36–38, 41, 42, 44], one involved stimulation of the common peroneal nerve [47], and one involved stimulation of the lower limb [41]. The most common stimulation site was the quadriceps.

Most studies had a high risk or some concerns regarding the overall risk of bias (S2–S5 Appendices).

### Primary outcomes

**Fig 2:**
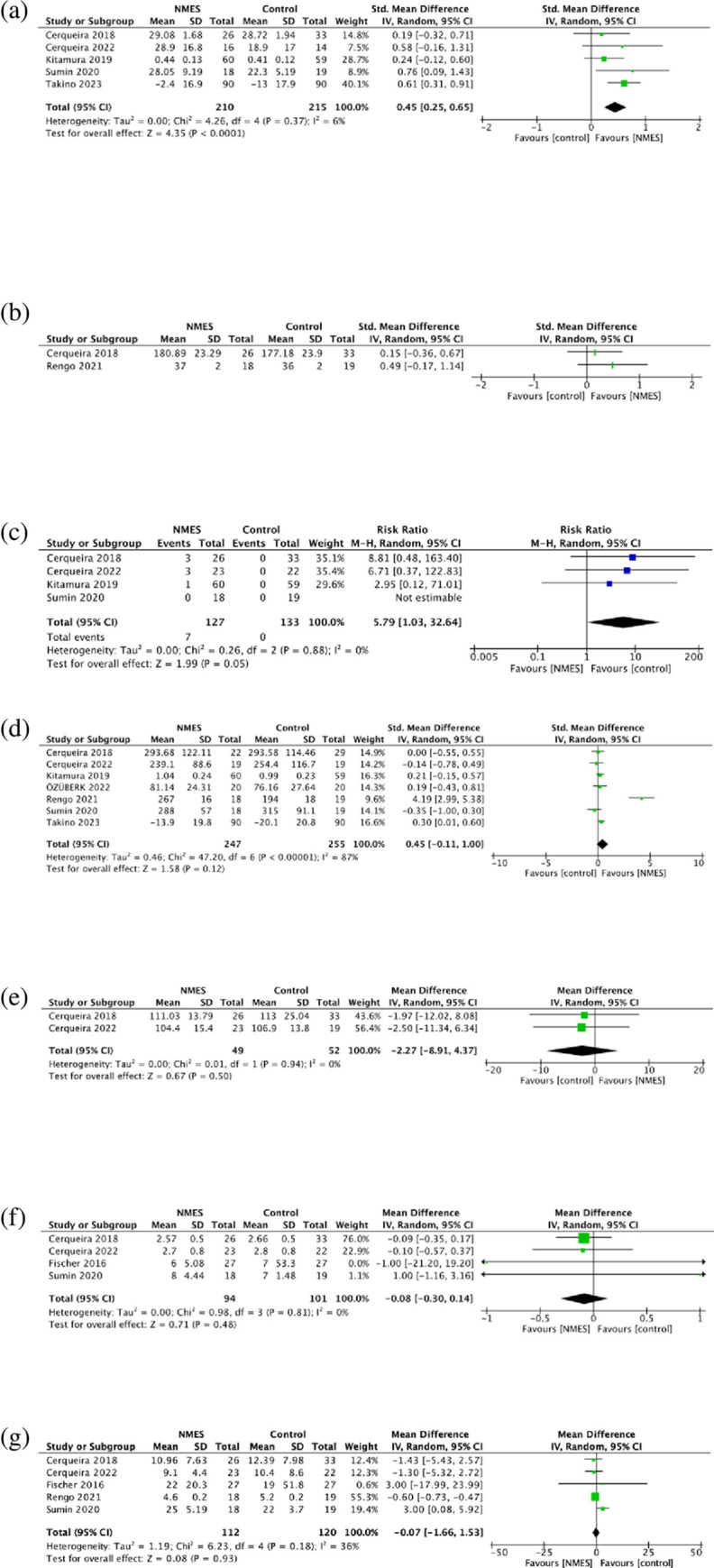
Forest plot of comparison. Cardiovascular Surgery (a) Lower limb muscle strength (b) HR-QOL (c) Adverse events (d) Walking ability (e) Activity of daily living (f) Length of stay in ICU (g) Length of stay in hospital.

The evidence suggested that NMES slightly increased lower limb muscle strength in cardiovascular surgery (five studies [30–39], 425 participants): SMD, 0.45, 95% CI 0.25 to 0.65; low certainty evidence (Fig 2A and Table 2). HR-QOL was measured in two trials [20, 23] in cardiovascular surgery. One study [34] used the Nottingham Health Profile, and the other [31] used SF-36 (Fig 2B), with very low certainty of evidence (Table 2). We decided not to conduct a meta-analysis because of the concept of heterogeneity with regard to HR-QOL. HR-QOL increased in both studies. NMES also slightly increased adverse events in cardiovascular surgery (four studies [32, 34, 35, 38], 260 participants): risk ratio 5.79; 95% CI 1.03 to 32.64, I^2^ = 0%; low certainty evidence (Fig 2C and Table 2). During one NMES application, three patients presented with hypotension [34, 38], three complained of pain [34, 38], and one complained of muscle discomfort [32] induced by NMES.

**Table 2:** Summary of findings 1. Summary of findings: Cardiovascular surgery.

**NMES compared to control for health problems in cardiovascular surgery**						
**Patient or population:** Health problems in cardiovascular surgery **Setting:** Hospital, community, or home settings **Intervention:** NMES **Comparison:** Control						
Outcomes	Anticipated absolute effects^*^ (95% CI)		Relative effect (95% CI)	№ of participants (studies)	Certainty of the evidence (GRADE)	Comments
	**Risk with Control**	**Risk with NMES**				
Lower limb muscle strength measured using MRC Lower limb and knee extensors strength. A higher score indicates higher lower limb muscle strength.	-	SMD **0.45 higher (0.25 higher to 0.65 higher)**	-	425 (5 RCTs)	⨁⨁◯◯ Low^a,b^	
HR-QOL measured using Nottingham Health Profile and SF-36. A higher score indicates higher HRQOL.	not pooled	not pooled	-	96 (2 RCTs)	⨁◯◯◯ Very low^a,c,d^	Only two studies reported data on HR-QOL and pooling of data was inappropriate due to differences in outcome measures. Individual study results are reported separately.
Adverse events	0 per 1,000	**50 per 1,000** **(0 to 110)**	**RR 5.79** **(1.03 to 32.64)**	260 (4 RCTs)	⨁⨁◯◯ Low^a,c^	
Walking ability measured using 6MWT (m), walking speed(m/sec). A higher score indicates faster walking.	-	SMD **0.45 higher** **(0.11 lower to 1.00 higher)**	-	502 (7 RCTs)	⨁◯◯◯ Very low^a,c,e^	
ADL measured using FIM A higher score indicates higher ADL.	-	MD 2.27 **lower** **(8.91 lower to 4.37 higher)**	-	101 (2 RCT)	⨁◯◯◯ Very low^a,b,c^	
Length of stay in ICU	-	MD **0.08 lower** **(0.30 lower to 0.14 higher)**	-	195 (4 RCTs)	⨁◯◯◯ Very low^a,c,f^	
Length of stay in hospital	-	MD **0.07 lower** **(1.66 lower to 1.53 higher)**	-	232 (5 RCTs)	⨁◯◯◯ Very low^a,c,g^	

*The risk in the intervention group (and its 95% confidence interval) is based on the assumed risk in the comparison group and the **relative effect** of the intervention (and its 95% CI).

**CI**: confidence interval; **MD:** mean difference; **RR:** risk ratio; **SMD:** standardized mean difference; **MRC:** Medical Research Council; **HR-QOL:** Health-related quality of life; **6MWT:** 6-Minute Walk Test; **ADL**: Activity of daily living; **FIM:** Functional Independence Measure; **ICU:** Intensive Care Unit

GRADE Working Group grades of evidence

**High certainty:** very confident that the true effect lies close to that of the estimate of the effect.

Moderate certainty: moderately confident in the effect estimate: the true effect is likely to be close to the estimate of the effect, but there is a possibility that it is substantially different.

Low certainty: confidence in the effect estimate is limited: the true effect may be substantially different from the estimate of the effect.

Very low certainty: very little confidence in the effect estimate: the true effect is likely to be substantially different from the estimate of effect.

Explanations

a. Downgraded one level for limitations in the study design.

b. Downgraded one level for imprecision reflecting small sample size

c. Downgraded one level for imprecision (wide CI)

d. Downgraded one level for inconsistency (heterogeneity of outcomes)

e. Downgraded one level for inconsistency (I^2^ = 87%).

f. Downgraded one level for inconsistency (point estimates vary widely among studies)

g. Downgraded one level for inconsistency (I^2^ = 36%).

For pulmonary surgery, lower limb muscle strength, HR-QOL, and adverse events were not reported.

**Fig 3:**
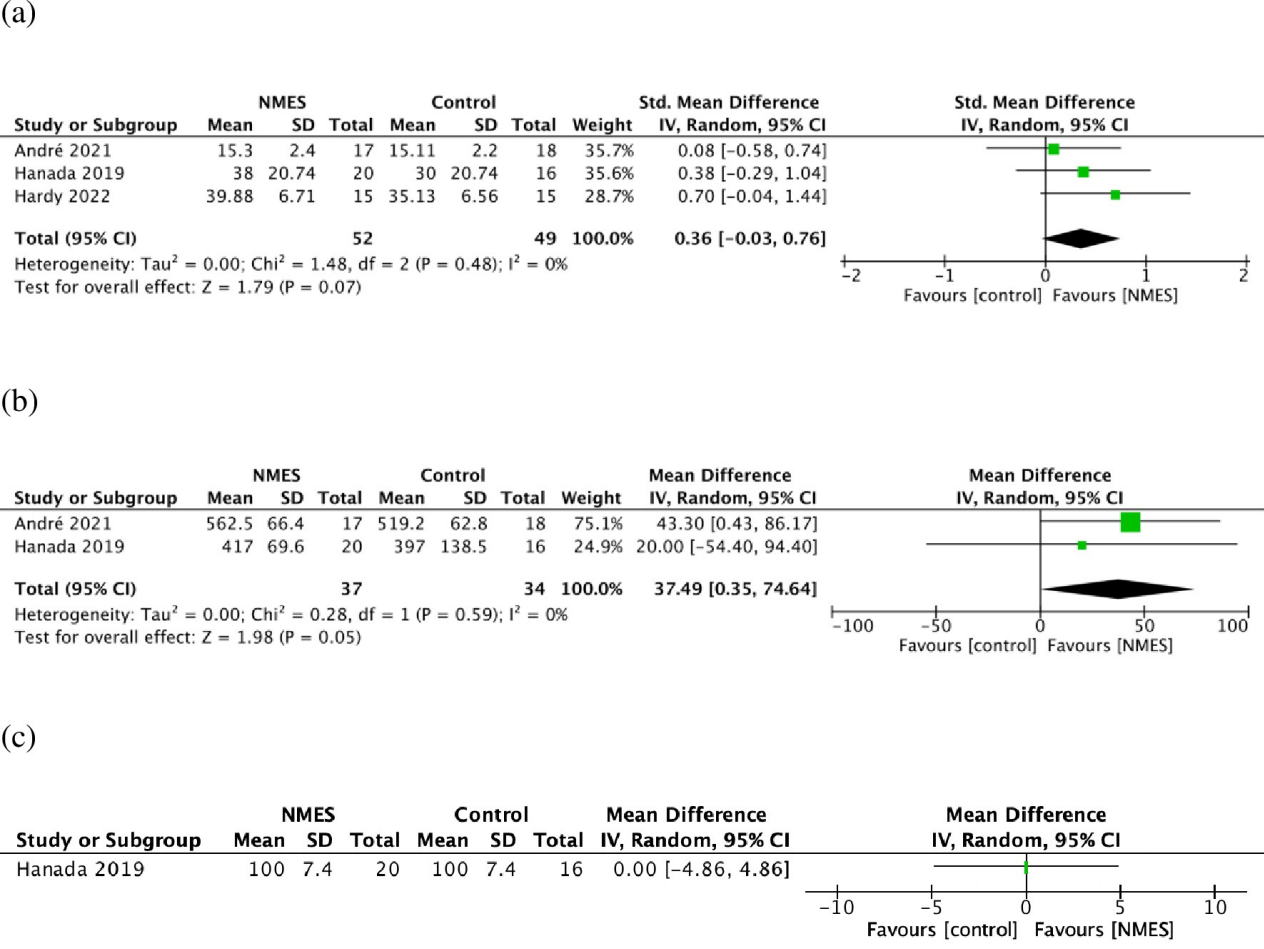
Forest plot of comparison. Digestive system surgery (a) Lower limb muscle strength (b) Walking ability (c) Activity of daily living.

The evidence is very uncertain about the effect of NMES on lower limb muscle strength in digestive system surgery (three studies [42, 43, 46], 86 participants): SMD, 0.36; 95% CI −0.03 to 0.76; very low certainty evidence (Fig 3A and Table 3). HR-QOL and adverse events were not reported for digestive system surgery.

**Table 3:** Summary of findings 2. Summary of findings: Digestive system surgery.

**NMES compared to control for health problems in digestive system surgery**					
**Patient or population:** Health problems in digestive system surgery **Setting:** Hospital, community, or home settings **Intervention:** NMES **Comparison:** Control					
Outcomes	Anticipated absolute effects^*^ (95% CI)	Relative effect (95% CI)	№ of participants (studies)	Certainty of the evidence (GRADE)
	**Risk with Control**				**Risk with NMES**
Lower limb muscle strength measured using quadriceps strength and isometric peripheral dominant knee muscle. A higher score indicates higher lower limb muscle strength.	-	SMD **0. 36 higher** **(0.03 lower to 0.76 higher)**	-	101 (3 RCTs)	⨁◯◯◯ Very low^a,b^
Adverse events	0 per 1,000	0 per 1,000	-	30 (1 RCT)	⨁◯◯◯ Very low^a,b^
Walking ability measured using 6MWT (m). A higher score indicates faster walking	-	MD **37.49 higher** **(0.35 higher to 74.64 higher)**	-	71 (2 RCT)	⨁◯◯◯ Very low^a,b^
Activity of daily living measured using BI. A higher score indicates higher ADL.	-	MD **0** **(4.86 lower to 4.86 higher)**	-	36 (1 RCT)	⨁◯◯◯ Very low^a,b^

*The risk in the intervention group (and its 95% confidence interval) is based on the assumed risk in the comparison group and the relative effect of the intervention (and its 95% CI).

**CI**: confidence interval; **MD:** mean difference; **SMD:** standardized mean difference; **6MWT:** 6-Minute Walk Test; **BI:** Barthel Index

GRADE Working Group grades of evidence

**High certainty:** very confident that the true effect lies close to that of the estimate of the effect.

Moderate certainty: moderately confident in the effect estimate: the true effect is likely to be close to the estimate of the effect, but there is a possibility that it is substantially different.

Low certainty: confidence in the effect estimate is limited: the true effect may be substantially different from the estimate of the effect.

Very low certainty: very little confidence in the effect estimate: the true effect is likely to be substantially different from the estimate of effect.

Explanations

a. Downgraded one level for limitations in the study design.

b. Downgraded two levels for imprecision (wide CI)

For other surgery, lower limb muscle strength, HR-QOL, and adverse events were not reported. The subgroups showed no significant differences in the primary outcomes in the prespecified subgroup analyses (S6 Appendix).

### Secondary outcomes

The evidence is very uncertain about the effect of NMES on walking ability in cardiovascular surgery (seven studies [31, 32, 34, 35, 37–39], 502 participants): SMD, 0.45, 95% CI −0.11 to 1.00; very low certainty evidence (Fig 2D and Table 2). NMES slightly increased ADL in cardiovascular surgery (two studies [34, 38], 101 participants): MD, -2.27, 95% CI −8.91 to 4.37; low certainty evidence (Fig 2E and Table 2). Additionally, the evidence is very uncertain about the effect of NMES on length of stay in the ICU in cardiovascular surgery (four studies [30, 34, 35, 38], 195 participants): MD, −0.08; 95% CI −0.30 to 0.14; very low certainty evidence (Fig 2F and Table 2). The evidence is very uncertain about the effect of NMES on the length of hospital stay in cardiovascular surgery (five studies [30, 31, 34, 35, 38], 232 participants): MD, 0.07, 95% CI -1.66 to 1.53; very low certainty evidence (Fig 2G and Table 2).

**Fig 4:**
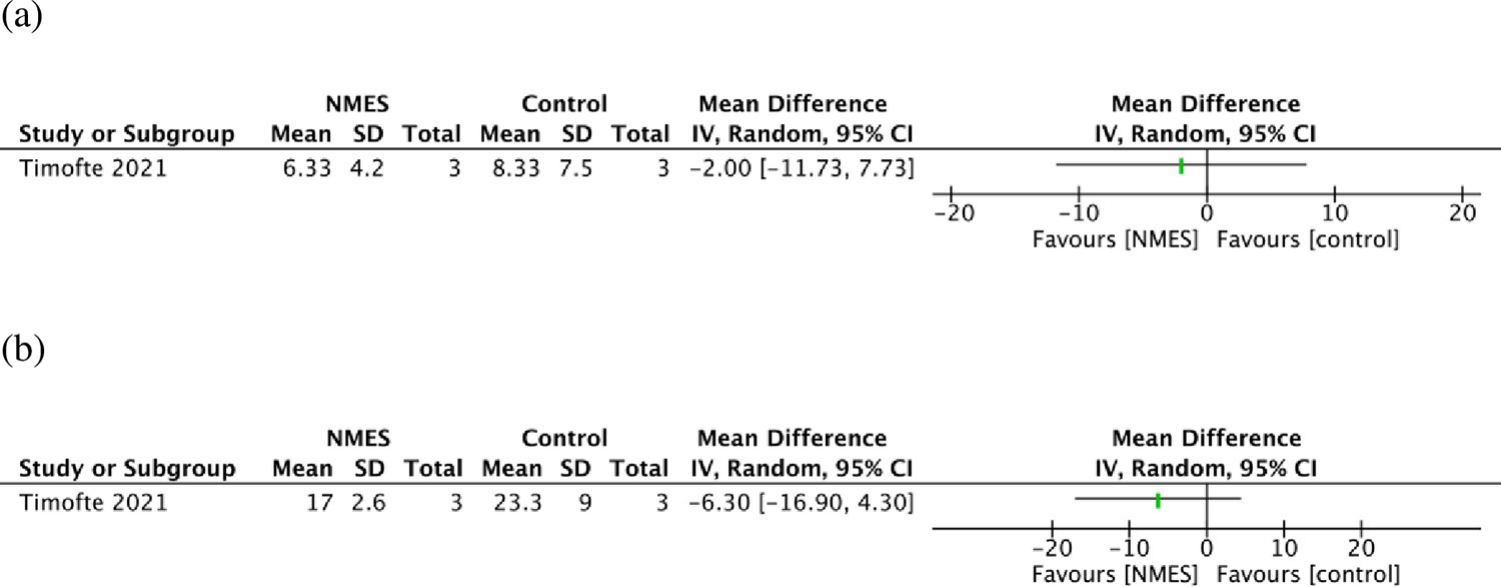
Forest plot of comparison. Pulmonary Surgery (a) Length of stay in ICU (b) Length of stay in hospital.

Furthermore, the evidence is very uncertain about the effect of NMES on length of stay in the ICU in pulmonary surgery (one study [40], 6 participants MD, −2.00, 95% CI −11.73–7.73; very low certainty evidence) (Fig 4A and Table 4). The evidence is very uncertain about the effect of NMES on the length of hospital stay in pulmonary surgery (one study [40], 6 participants: MD, −6.30, 95% CI −16.90 to 4.30; very low certainty evidence) (Fig 4B and Table 4).

**Table 4:** Summary of findings 2. Summary of findings: Pulmonary surgery.

**NMES compared to controls for health problems in pulmonary surgery**					
**Patient or population:** Health problems in pulmonary surgery **Setting:** hospital **Intervention:** NMES **Comparison:** Control					
Outcomes	Anticipated absolute effects^*^ (95% CI)		Relative effect (95% CI)	№ of participants (studies)	Certainty of the evidence (GRADE)
	**Risk with Control**	**Risk with NMES**			
Length of stay in ICU	-	MD 2 lower (11.73 lower to 7.73 higher)	-	6 (1 RCT)	⨁◯◯◯ Very low^a,b^
Length of stay in hospital	-	MD 6.3 lower (16.9 lower to 4.3 higher)	-	6 (1 RCT)	⨁◯◯◯ Very low^a,b^

*The risk in the intervention group (and its 95% confidence interval) is based on the assumed risk in the comparison group and the **relative effect** of the intervention (and its 95% CI).

CI: confidence interval; **MD:** mean difference

GRADE Working Group grades of evidence

**High certainty:** confident that the true effect is close to the effect estimate.

Moderate certainty: moderately confident in the effect estimate: the true effect is likely to be close to the estimate of the effect, but there is a possibility that it is substantially different.

Low certainty: confidence in the effect estimate is limited: the true effect may be substantially different from the estimate of the effect.

Very low certainty: very little confidence in the effect estimate: the true effect is likely to be substantially different from the estimate of effect.

Explanations

a. Downgraded one level for limitations in the study design.

b. Downgraded two levels for imprecision (wide CI).

In terms of digestive system surgery, the evidence was very uncertain about the effect of NMES on walking ability (two studies [42, 43], 71 participants: MD, 37.49, 95% CI 0.35 to 74.64; very low certainty evidence) (Fig 3B and Table 3) and ADL (one study [43], 36 participants): MD, 0.00, 95% CI −4.86 to 4.86; very low certainty evidence (Fig 3C and Table 3).

**Fig 5:**
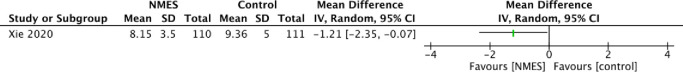
Forest plot of comparison: Other surgery (length of stay in hospital).

Moreover, The evidence suggests that NMES slightly reduced the length of hospital stay in other surgeries (one study [47], 221 participants): MD, −1.21, 95% CI −2.35 −0.07; low certainty evidence (Fig 5 and Table 5).

**Table 5:** Summary of findings 4. Summary of findings: Other surgeries.

**NMES compared to the control for health problems in other surgery**					
**Patient or population:** Health problems in other surgery **Setting:** hospital **Intervention:** NMES **Comparison:** Control					
Outcomes	Anticipated absolute effects^*^ (95% CI)		Relative effect (95% CI)	№ of participants (studies)	Certainty of the evidence (GRADE)
	**Risk with Control**	**Risk with NMES**			
Length of stay in hospital	-	MD **1.21 lower** **(2.35 lower to 0.07 lower)**	-	221 (1 RCT)	⨁⨁◯◯ Low^a,b^

*The risk in the intervention group (and its 95% confidence interval) is based on the assumed risk in the comparison group and the **relative effect** of the intervention (and its 95% CI).

CI: confidence interval; **MD:** mean difference

GRADE Working Group grades of evidence

**High certainty:** confident that the true effect is close to the effect estimate.

Moderate certainty: moderately confident in the effect estimate: the true effect is likely to be close to the estimate of the effect, but there is a possibility that it is substantially different.

Low certainty: confidence in the effect estimate is limited: the true effect may be substantially different from the estimate of the effect.

Very low certainty: very little confidence in the effect estimate: the true effect is likely to be substantially different from the estimate of effect.

Explanations

a. Downgraded one level for limitations in the study design

b. Downgraded one level for imprecision (wide CI)

## Discussion

We observed a slight increase in lower limb muscle strength in cardiovascular surgery patients undergoing postoperative NMES, with little increase in adverse events. Postoperative NMES in pulmonary, digestive system, and other surgeries, have rarely been studied. The results of this systematic review indicate that the certainty of the evidence is very low.

We clarified the efficacy and adverse events of NMES following cardiovascular surgery more precisely than in a previous systematic review [13]. In the previous study, one quasi-RCT was included in a meta-analysis of knee extensor strength [48]. We excluded this study to investigate its precise efficacy. The results showed a slight increase in lower limb muscle strength. In addition, we examined the adverse events. Seven patients (5%) experienced minor adverse events (hypotension in three, pain in three, and muscle discomfort in one patient). A previous RCT including critically ill patients reported that after the first NMES session, one patient (7%) experienced superficial burns due to incorrect stimulation mode settings [49]. Additionally, a previous prospective observational study, which included 11 critically ill patients, reported that no patients experienced adverse events [50]. This suggests that NMES intervention slightly increases lower limb muscle strength following cardiovascular surgery without serious complications, although burns should be noted.

Reports on lower limb muscle strength, HR-QOL, and adverse events are lacking on pulmonary, digestive systems and other surgeries. Lower limb muscle strength is reportedly associated with mortality [51], and HR-QOL is considered an important core outcome [52, 53]. Therefore, studies including lower limb muscle strength and HR-QOL as outcomes are needed. Lower limb muscle strength and walking ability decrease after surgery for esophageal [54] and pancreatic cancer [55], and it has been reported that decreased physical function is associated with lower HR-QOL. However, no studies have been conducted on patients with esophageal or pancreatic cancer. We consider that a larger, well-designed RCT, including a core outcome set such as HR-QOL is needed for pulmonary and digestive system surgery (especially esophageal and pancreatic cancer).

For other surgeries, there was only one RCT (kidney transplantation), which may have reduced the length of hospital stay, a secondary outcome of this study. However, the primary outcomes of this study: lower limb muscle strength, HR-QOL, and adverse events were not reported. Moreover, a recent review of the effects of NMES in ICU patients stated that it was effective in improving muscle strength and reducing the length of hospital stay [56]. Although the potential to increase lower limb muscle strength in kidney transplantation patients need to be studied, clinicians should consider NMES intervention after kidney transplantation.

For cardiovascular surgery, evidence regarding the effects of NMES on walking ability, ADL, length of stay in ICU, and length of hospital stay, compared with the effects of usual care, was very uncertain. For digestive system surgery, the effects of NMES on walking ability and ADL were very uncertain. These results were largely influenced by the limitations in the study design, the small sample size, and the inconsistent results. Therefore, well-designed RCTs with large sample sizes are necessary.

This review has several strengths. First, we used a strict methodology that adhered to a written protocol created beforehand following the PRISMA 2020 statement, including an extensive search of supporting data. Second, to the best of our knowledge, this is the first systematic review of postoperative NMES interventions for thoracic and abdominal surgery incorporating the pulmonary system, digestive system, and other surgeries.

Nevertheless, this systematic review has some limitations. First, all outcomes had low to very low certainty of evidence; therefore, the fact that the certainty of the evidence was low or very low should be interpreted with caution while interpreting the results. Second, the controls in this study were managed in various ways, including no treatment, sham interventions, usual rehabilitation, and routine care. Differences in control group management may have led to differences in effectiveness.

## Conclusions

Clinicians should consider NMES interventions for patients who undergo cardiovascular surgery since it slightly increases lower limb muscle strength with only little increase in adverse events. Larger, well-designed RCTs that include important outcomes such as HR-QOL and adverse events are needed to investigate the effectiveness of NMES interventions in patients who undergo thoracic and abdominal surgeries, including cardiovascular, pulmonary system, digestive system, and other surgeries.

## Supporting information

S1 Table PRISMA 2020 checklist. (PDF)

S2 Table Reasons for exclusion of 27 studies. (PDF)

S1 Appendix Search strategy. (PDF)

S2 Appendix Risk of bias summary: Cardiovascular surgery (a) Lower limb muscle strength (b) HR-QOL (c) Adverse events (d) Walking ability (e) Activity of daily living (f) Length of stay in ICU (g) Length of stay in hospital.
(PDF)

S3 Appendix Risk of bias summary: Pulmonary surgery (a) Length of stay in ICU (b) Length of stay in hospital.
(PDF)

S4 Appendix Risk of bias summary: Digestive system surgery (a) Lower limb muscle strength (b) Walking ability (c) Activity of daily living.
(PDF)

S5 Appendix Risk of bias summary: Other surgery (length of stay in hospital). (PDF)

S6 Appendix Subgroup analysis: Cardiovascular surgery (lower limb muscle strength). (PDF)
