# Supplementary material for: Efficacy of neuromuscular electrical stimulation for thoracic and abdominal surgery: A systematic review and meta-analysis
Source: PLoS One. 2023 Nov 30;18(11):e0294965. doi: 10.1371/journal.pone.0294965 (PMC10688715; doi:10.1371/journal.pone.0294965)
Supplement: S2 Table — (PDF) [file pone.0294965.s002.pdf]

S3. Reasons for exclusion of 27 studies

Title	First author	Year	Exclusion Reasons	Digital Object Identifier (DOI) or URL
Coordinated increase in skeletal muscle fiber area and expression of IGF-I with resistance exercise in elderly post-operative patients	Suetta C	2010	wrong population	10.1016/j.ghir.2009.11.005
Curative efficacy of low frequency electrical stimulation in preventing urinary retention after cervical cancer operation	Li H	2019	wrong intervention	10.1186/s12957-019-1689-2
Effects of muscle electrical stimulation on peak VO2 in cardiac transplant patients	Vaquero AF	1998	wrong population	10.1055/s-2007-971924
Effects of neuromuscular electrical stimulation on muscle layer thickness of knee extensor muscles in intensive care unit patients: a pilot study	Gruther W	2010	wrong population	10.2340/16501977-0564
Functional electrical stimulation improves muscle strength and endurance in patients after cardiac surgery: a randomized controlled trial	Schardong J	2017	wrong population	10.1016/j.bjpt.2017.05.004
Neuromuscular Electrical Stimulation and Strength Recovery of Postnatal Diastasis Recti Abdominis Muscles	Dalia M Kamel	2017	wrong population	10.5535/arm.2017.41.3.465
Neuromuscular electrical stimulation as a possible means to prevent muscle tissue wasting in artificially ventilated and sedated patients in the intensive care unit: a pilot study	Meesen RL	2010	wrong population	10.1111/j.1525-1403.2010.00294.x
Neuromuscular electrical stimulation: no enhancement of recovery from maximal exercise	Malone JK	2014	wrong population	10.1123/ijspp.2013-0327
Effects of transcutaneous electrical nerve stimulation in abdominal postoperative patients: A prospective randomized single-blind study	Mitsunori Tokuda	2012	wrong intervention	https://upload.umin.ac.jp/cgi-open-bin/ctr_e/ctr_view.cgi?recptno=R000009473
Electrostimulation of Skeletal Muscles in Patients Listed for a Heart Transplant	Andrey Bezdenezhnykh	2020	wrong population	https://clinicaltrials.gov/show/NCT04522609
Feasibility of electrical muscle stimulation in long-term mechanically ventilated patients in medical-surgical intensive care unit: experience from a Canadian centre	Sunita Mathur	2014	wrong population	https://www.cochranelibrary.com/central/doi/10.1002/central/CN-01106908/full
ICEAGE (Incidence of Complications following Emergency Abdominal surgery: Get Exercising):	Ianthe Boden	2015	wrong intervention	https://www.anzctr.org.au/Trial/Registration/TrialReview.aspx?id=365543

a pragmatic, multicentre, randomised controlled trial testing an enhanced physiotherapy program for the prevention of complications and improved physical recovery after emergency abdominal surgery.				
Implementing lift system to physical therapy program for early mobilization in immobilised patients: A randomised controlled trial	Tomris Duymaz	2018	wrong population	http://www.anzctr.org.au/ACTRN12618001336279.aspx
Prehabilitation for Cardiac Surgery in Patients With Reduced Exercise Tolerance	Andrey V Bezdenezhnykh	2020	wrong population	https://clinicaltrials.gov/ct2/show/NCT04545268
Randomized controlled pilot study of neuromuscular electrical stimulation of the quadriceps in patients with non-small cell lung cancer	Maddocks M	2009	wrong population	10.1016/j.jpainsymman.2009.05.011
Venous Blood Flow Velocity: Electrical Foot Stimulation Compared to Intermittent Pneumatic Compression of the Foot	Robert E Kaplan	2005	wrong population	https://clinicaltrials.gov/ct2/show/NCT00114608
Comparative study of the effect of neuromuscular electrical stimulation and oral administration of branched-chain amino acid on preventing sarcopenia in patients after living-donor liver transplantation: study protocol for an open-label randomized controlled trial	Haraguchi M	2021	wrong intervention	10.1186/s13063-021-05086-y
Effects of Functional Electrical Stimulation in Patients Undergoing Coronary Artery Bypass Grafting Surgery	Isabella Martins de Albuquerque	2018	wrong population	https://clinicaltrials.gov/ct2/show/NCT03560713
Effects of Functional Electrical Stimulation on Physical Performance of Patients in Cardiac Rehabilitation	Antônio Marcos Vargas da Silva	2014	wrong population	https://clinicaltrials.gov/show/NCT02088138
Effects of neuromuscular electrical stimulation of quadriceps force and oral administration of branched-chain amino acid in patients after Living-donor liver transplantation - Effects of neuromuscular electrical stimulation and branched chain amino acid in patients after Living-donor liver transplantation	Hisamitsu Miyaaki	2019	wrong intervention	https://upload.umin.ac.jp/cgi-open-bin/icdr_e/ctr_view.cgi?recptno=R000040949
Implementation of an Early Rehabilitation Program for the Patient With Lung Transplantation: From the ICU to Home.	Generalitat de Catalunya	2020	wrong population	https://clinicaltrials.gov/ct2/show/NCT04244734
The Role of Transcutaneous Electrical Nerve Stimulation Combined With Physiotherapy and Rehabilitation Program on Pulmonary Function and Functional Exercise Capacity in Patients	Aysel Yildiz Ozer	2009	wrong intervention	https://clinicaltrials.gov/ct2/show/NCT04879108

Undergoing Thoracic Surgery? A Randomized Controlled Trial				
Use of neuromuscular electrical stimulation to preserve the thickness of abdominal and chest muscles of critically ill patients: a randomized clinical trial	Dall' Acqua AM	2017	wrong intervention	10.2340/16501977-2168
Functional electrical stimulation associated with combined post-CABG training: a randomized clinical trial	Nubia Gonzatti	2021	wrong population	10.1590/1809-2950/20031628012021
Early Neuromuscular Electrical Stimulation in Addition to Early Mobilization Improves Functional Status and Decreases Hospitalization Days of Critically Ill Patients	Campos, Débora R	2022	wrong population	http://dx.doi.org/10.1097/CCM.0000000000005557
Study protocol for neuromuscular stimulation for rehabilitation after general and vascular surgery: a pilot randomised clinical study	Nimura Megumi	2023	wrong population	http://dx.doi.org/10.1136/bmjopen-2022-061800
Neuromuscular Electrical Stimulation After Lung Transplantation.	Graciele Sbruzzi	2018	wrong intervention	https://clinicaltrials.gov/ct2/show/NCT03788876.ClinicalTrials.gov .
