# Supplementary material for: Efficacy of neuromuscular electrical stimulation for thoracic and abdominal surgery: A systematic review and meta-analysis
Source: PLoS One. 2023 Nov 30;18(11):e0294965. doi: 10.1371/journal.pone.0294965 (PMC10688715; doi:10.1371/journal.pone.0294965)
Supplement: S1 Appendix — (PDF) [file pone.0294965.s003.pdf]

S2 Appendix
MEDLINE (Ovid) search strategy

1	exp Digestive System Surgical Procedures/ or exp Cardiovascular Surgical Procedures/ or exp Thoracic Surgical Procedures/ or exp Urogenital Surgical Procedures/ or exp General Surgery/ or exp Colorectal Surgery/ or exp Gynecology/ or exp Urology/ or exp Surgical Oncology/ or exp Thoracic Surgery/ or exp Laparotomy/ or exp Perioperative Period/ or exp Pneumonectomy/ or exp Organ Transplantation/
2	((heart or cardi$ or valve or aort$ or mitral or pulmonary or tricuspid or congenital defect$ or gastrointestinal or urologic$ or abdo$ or general or gynecologic$ or esophag$ or lung or colo$ or rectal or pancre$ or baria$ or liver or hepatocellular or gastr$ or stomach) adj3 (surg$ or operat$ or procedure$ or transplan$ or rep$ or annuloplasty)).mp.
3	(cabg or maze).mp.
4	exp Electric Stimulation Therapy/
5	exp Electric stimulation/
6	(electrotherap* or (electric$ adj3 stimulation) or electromyostimulation or electrostimulation or (neuromuscular adj3 stimulation) or (neuromuscular adj3 electric*) or EMS or NMES).mp.
7	or/1-3
8	or/4-6
9	randomized controlled trial.pt.
10	controlled clinical trial.pt.
11	randomized.ab.
12	placebo.ab.
13	drug therapy.fs.
14	randomly.ab.
15	trial.ab.
16	groups.ab.
17	or/9-16
18	exp animals/ not humans.sh.
19	17 not 18
20	7 and 8 and 19
21	randomized controlled trial.pt.
22	controlled clinical trial.pt.
23	randomized.ab.
24	placebo.ab.
25	clinical trials as topic.sh.
26	randomly.ab.
27	trial.ti.
28	or/21-27
29	exp animals/ not humans.sh.

30	28 not 29
31	7 and 8 and 30

CENTRAL (Cochrane Library) search strategy

1	[mh "Digestive System Surgical Procedures"] OR [mh "Cardiovascular Surgical Procedures"] OR [mh "Thoracic Surgical Procedures"] OR [mh "Urogenital Surgical Procedures"] OR [mh "General Surgery"] OR [mh "Colorectal Surgery"] OR [mh Gynecology] OR [mh Urology] OR [mh "Surgical Oncology"] OR [mh "Thoracic Surgery"] OR [mh Laparotomy] OR [mh "Perioperative Period"] OR [mh Pneumonectomy] OR [mh "Organ Transplantation"]
2	((heart:ti,ab,kw OR cardi?:ti,ab,kw OR valve:ti,ab,kw OR aort?:ti,ab,kw OR mitral:ti,ab,kw OR pulmonary:ti,ab,kw OR tricuspid:ti,ab,kw OR ("congenital" NEXT defect?):ti,ab,kw OR gastrointestinal:ti,ab,kw OR urologic?:ti,ab,kw OR abdo?:ti,ab,kw OR general:ti,ab,kw OR gynecologic?:ti,ab,kw OR esophag?:ti,ab,kw OR lung:ti,ab,kw OR colo?:ti,ab,kw OR rectal:ti,ab,kw OR pancre?:ti,ab,kw OR baria?:ti,ab,kw OR liver:ti,ab,kw OR hepatocellular:ti,ab,kw OR gastr?:ti,ab,kw OR stomach:ti,ab,kw) NEAR/3 (surg?:ti,ab,kw OR operat?:ti,ab,kw OR procedure?:ti,ab,kw OR transplan?:ti,ab,kw OR rep?:ti,ab,kw OR annuloplasty:ti,ab,kw))
3	(cabg:ti,ab,kw OR maze:ti,ab,kw)
4	[mh "Electric Stimulation Therapy"] OR [mh "Electric stimulation"]
5	(electrotherap*:ti,ab,kw OR (electric?:ti,ab,kw NEAR/3 stimulation:ti,ab,kw) OR electromyostimulation:ti,ab,kw OR electrostimulation:ti,ab,kw OR (neuromuscular:ti,ab,kw NEAR/3 stimulation:ti,ab,kw) OR (neuromuscular:ti,ab,kw NEAR/3 electric*:ti,ab,kw) OR EMS:ti,ab,kw OR NMES:ti,ab,kw)
6	#1 OR #2 OR #3
7	#4 OR #5
8	#6 AND #7

EMBASE (Dialog) search strategy

Set#	Searched for
S1	EMB.EXACT.EXPLODE("cardiovascular surgery")
S2	EMB.EXACT.EXPLODE("thorax surgery")
S3	EMB.EXACT.EXPLODE("urologic surgery")
S4	EMB.EXACT.EXPLODE("general surgery")
S5	EMB.EXACT.EXPLODE("colorectal surgery")
S6	EMB.EXACT.EXPLODE("gynecology")
S7	EMB.EXACT.EXPLODE("urology")
S8	EMB.EXACT.EXPLODE("surgical oncology")
S9	EMB.EXACT.EXPLODE("laparotomy")
S10	EMB.EXACT.EXPLODE("perioperative period")
S11	EMB.EXACT.EXPLODE("organ transplantation")
S12	S1 OR S2 OR S3 OR S4 OR S5 OR S6 OR S7 OR S8 OR S9 OR S10 OR S11
S13	ab((heart OR cardi* OR valve OR aort* OR mitral OR pulmonary OR tricuspid OR "congenital defect*" OR gastrointestinal OR urologic* OR abdo* OR general OR gynecologic* OR esophag* OR lung OR colo* OR rectal OR pancre* OR baria* OR liver OR hepatocellular OR gastr* OR stomach) PRE/3 (surg* OR operat* OR procedure* OR transplan* OR rep*

	OR annuloplasty))
S14	ti((heart OR cardi* OR valve OR aort* OR mitral OR pulmonary OR tricuspid OR "congenital defect*" OR gastrointestinal OR urologic* OR abdo* OR general OR gynecologic* OR esophag* OR lung OR colo* OR rectal OR pancre* OR baria* OR liver OR hepatocellular OR gastr* OR stomach) PRE/3 (surg* OR operat* OR procedure* OR transplan* OR rep* OR annuloplasty))
S15	ab(CABG) OR ti(CABG)
S16	ab(Maze) OR ti(Maze)
S17	S13 OR S14 OR S15 OR S16
S18	EMB.EXACT.EXPLODE("electrotherapy")
S19	EMB.EXACT.EXPLODE("electrostimulation")
S20	ab((electrotherap* OR (electric* PRE/3 stimulation) OR electromyostimulation OR electrostimulation OR (neuromuscular PRE/3 stimulation) OR (neuromuscular PRE/3 electric*) OR EMS OR NMES))
S21	ti((electrotherap* OR (electric* PRE/3 stimulation) OR electromyostimulation OR electrostimulation OR (neuromuscular PRE/3 stimulation) OR (neuromuscular PRE/3 electric*) OR EMS OR NMES))
S22	(S12 OR S17) AND (S18 OR S19 OR S20 OR S21)
S23	(ab(random*) OR ti(random*)) OR (ab(clinical NEAR/1 trial*) OR ti(clinical NEAR/1 trial*)) OR (EMB.EXACT("health care quality"))
S24	S22 AND S23
S25	(ab(random*) OR ti(random*)) OR (ab(placebo*) OR ti(placebo*)) OR (ab(double NEAR/1 blind*) OR ti(double NEAR/1 blind*))
S26	S22 AND S25

PEDro search strategy
Advance search
Method: clinical trial Therapy: electrotherapies, heat, cold
Abstract & Title: heart OR cardio OR cardiac OR valve OR mitral OR pulmonary OR tricuspid OR congenital OR gastrointestinal OR urologic OR Abdominal OR general OR gynecologic OR esophageal OR lung OR colon OR colorectal OR rectal OR pancreas OR pancreatic OR liver OR hepatocellular OR gastric OR stomach OR CABG OR Maze

CINAHL search strategy

1	(MH "Digestive System Surgical Procedures"+) OR (MH "Cardiovascular Surgical Procedures"+) OR (MH "Thoracic Surgical Procedures"+) OR (MH "Urogenital Surgical Procedures"+) OR (MH "General Surgery"+) OR (MH "Colorectal Surgery"+) OR (MH Gynecology+) OR (MH Urology+) OR (MH "Surgical Oncology"+) OR (MH "Thoracic Surgery"+) OR (MH Laparotomy+) OR (MH "Perioperative Period"+) OR (MH Pneumonectomy+) OR (MH "Organ Transplantation"+)
2	((heart OR cardi? OR valve OR aort? OR mitral OR pulmonary OR tricuspid OR "congenital defect?" OR gastrointestinal OR urologic? OR abdo? OR general OR gynecologic? OR esophag? OR lung OR colo? OR rectal OR pancre? OR baria? OR liver OR hepatocellular OR gastr? OR stomach) N3 (surg? OR operat? OR procedure? OR transplan? OR rep? OR annuloplasty))
3	(cabg OR maze)
4	(MH "Electric Stimulation Therapy"+)
5	(MH "Electric stimulation"+)
6	(electrotherap* OR (electric? N3 stimulation) OR electromyostimulation OR electrostimulation OR (neuromuscular N3 stimulation) OR (neuromuscular N3 electric*) OR EMS OR NMES)
7	1 OR 2 OR 3
8	4 OR 5 OR 6
9	7 AND 8
10	((MH randomized controlled trials) OR (MH double-blind studies) OR (MH single-blind studies) OR (MH random assignment) OR (MH pretest-posttest

	design) OR (MH cluster sample) OR (TI (randomised OR randomized)) OR (AB (random*)) OR (TI (trial)) OR (MH (sample size) AND AB (assigned OR allocated OR control)) OR (MH (placebos)) OR (PT (randomized controlled trial)) OR (AB (control W5 group)) OR (MH (crossover design) OR MH (comparative studies)) OR (AB (cluster W3 RCT))) NOT (((MH animals+) OR (MH (animal studies)) OR (TI (animal model*))) NOT (MH (human)))
11	9 AND 10

ICTRP search strategy

advanced search

#1 Conditions: ((heart OR cardio OR cardiac OR valve OR mitral OR pulmonary OR tricuspid OR “congenital defect” OR gastrointestinal OR urologic OR urological OR abdomen OR abdominal OR gynecological OR gynecologic OR esophageal OR lung OR colon OR colorectal OR rectal OR pancreas OR pancreatic OR bariatric OR liver OR hepatocellular OR gastric OR gastrectomy OR stomach)) AND (surgery OR surgical OR operate OR operation OR operative OR procedure OR procedures OR transplant OR transplantation OR repair OR replacement OR annuloplasty) OR MAZE OR CABG

#2 Intervention: (electric OR electro OR stimulation OR stimulate OR neuromuscular OR NMES OR EMS)

#3 #1 AND #2

Recruitment status is ALL.

ClinicalTrials.gov search strategy

Condition or disease: ((heart OR cardio OR cardiac OR valve OR mitral OR pulmonary OR tricuspid OR “congenital defect” OR gastrointestinal OR urologic OR urological OR abdomen OR abdominal OR gynecological OR gynecologic OR esophageal OR lung OR colon OR colorectal OR rectal OR pancreas OR pancreatic OR bariatric OR liver OR hepatocellular OR gastric OR gastrectomy OR stomach)) AND (surgery OR surgical OR operate OR operation OR operative OR procedure OR procedures OR transplant OR transplantation OR repair OR replacement OR annuloplasty) OR MAZE OR CABG

Intervention: (electric OR electro OR stimulation OR stimulate OR neuromuscular OR NMES OR EMS)

Eligibility Criteria: Age:18+
