# Supplementary material for: Efficacy of neuromuscular electrical stimulation for thoracic and abdominal surgery: A systematic review and meta-analysis
Source: PLoS One. 2023 Nov 30;18(11):e0294965. doi: 10.1371/journal.pone.0294965 (PMC10688715; doi:10.1371/journal.pone.0294965)
Supplement: S2 Appendix — Risk of bias summary: Cardiovascular surgery (a) Lower limb muscle strength (b) HR-QOL (c) Adverse events (d) Walking ability (e) Activity of daily living (f) Length of stay in ICU (g) Length of stay in hospital. (PDF) [file pone.0294965.s004.pdf]

S4 Appendix: Risk of bias summary: Cardiovascular surgery (a) Lower limb muscle strength (b) HR-QOL (c) Adverse events (d) Walking ability (e) Activity of daily living (f) Length of stay in ICU (g) Length of stay in hospital

(a)

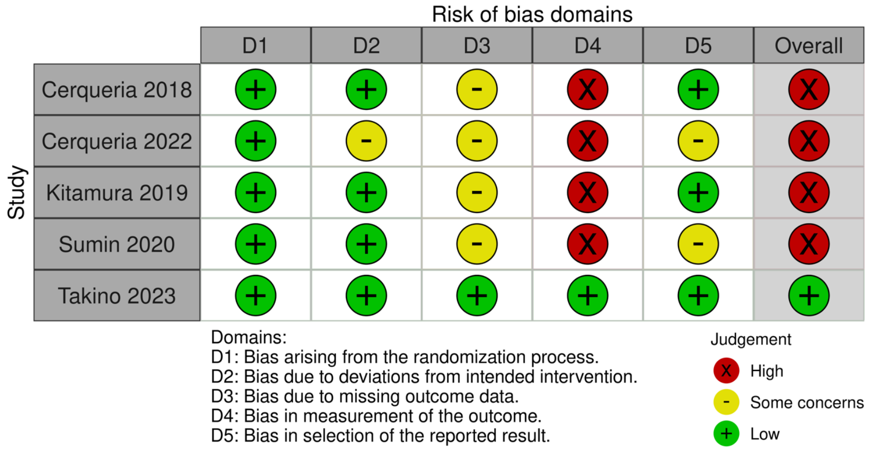

(b)

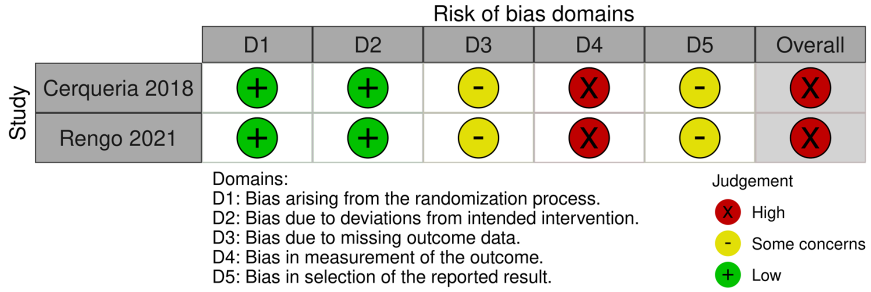

(c)

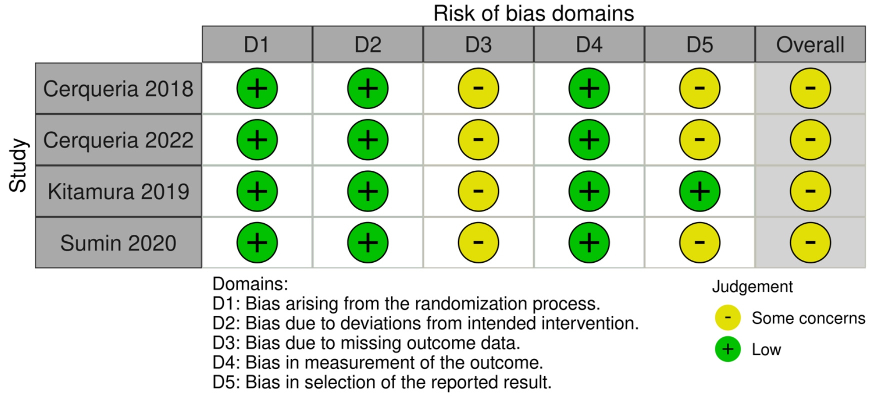

(d)

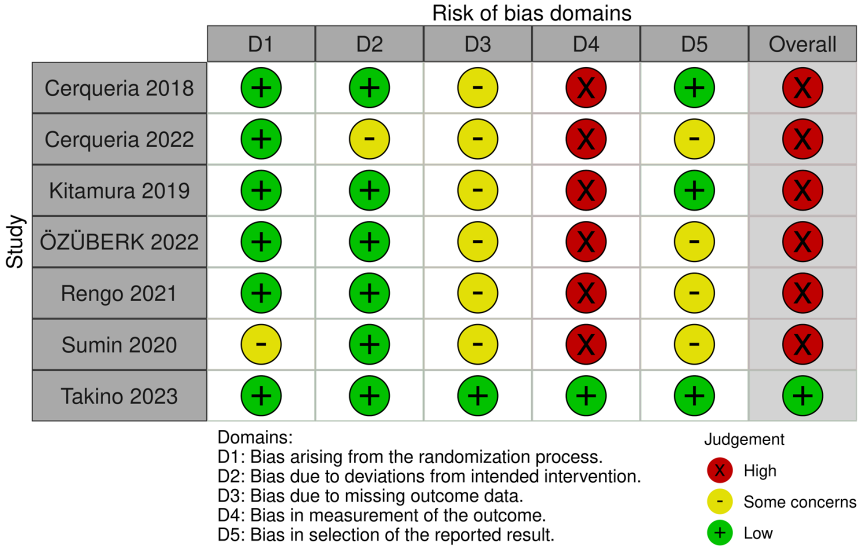
Risk of bias domains
D1
D2
D3
D4
D5
Overall
Study
Cerqueria 2018
Cerqueria 2022
Kitamura 2019
ÖZÜBERK 2022
Rengo 2021
Sumin 2020
Takino 2023
Domains:
D1: Bias arising from the randomization process.
D2: Bias due to deviations from intended intervention.
D3: Bias due to missing outcome data.
D4: Bias in measurement of the outcome.
D5: Bias in selection of the reported result.
Judgement
High
Some concerns
Low

(e)

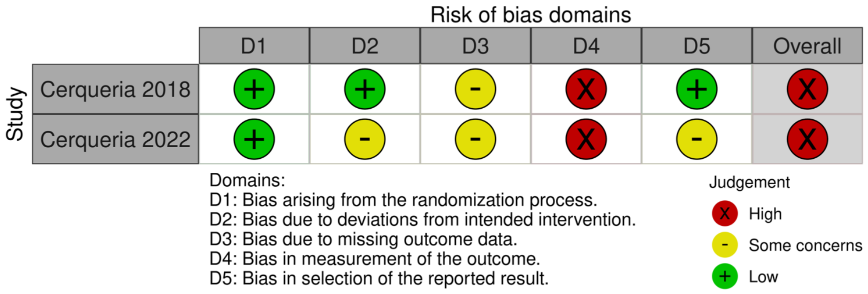
Risk of bias domains
D1
D2
D3
D4
D5
Overall
Study
Cerqueria 2018
Cerqueria 2022
Domains:
D1: Bias arising from the randomization process.
D2: Bias due to deviations from intended intervention.
D3: Bias due to missing outcome data.
D4: Bias in measurement of the outcome.
D5: Bias in selection of the reported result.
Judgement
High
Some concerns
Low

(f)

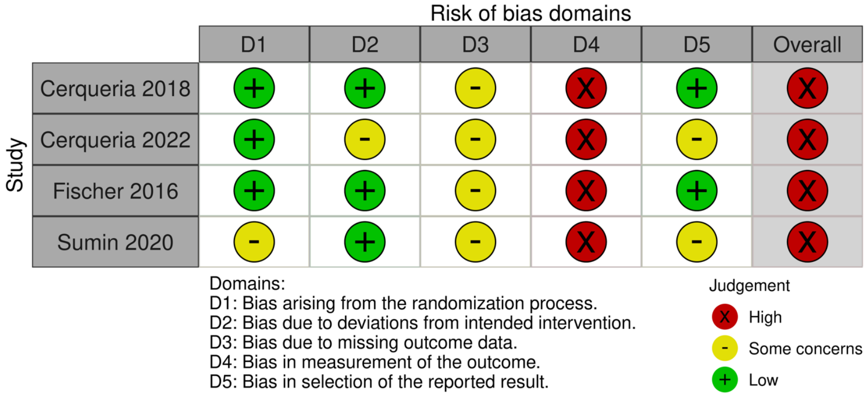
Risk of bias domains
D1
D2
D3
D4
D5
Overall
Study
Cerqueria 2018
Cerqueria 2022
Fischer 2016
Sumin 2020
Domains:
D1: Bias arising from the randomization process.
D2: Bias due to deviations from intended intervention.
D3: Bias due to missing outcome data.
D4: Bias in measurement of the outcome.
D5: Bias in selection of the reported result.
Judgement
High
Some concerns
Low

(g)

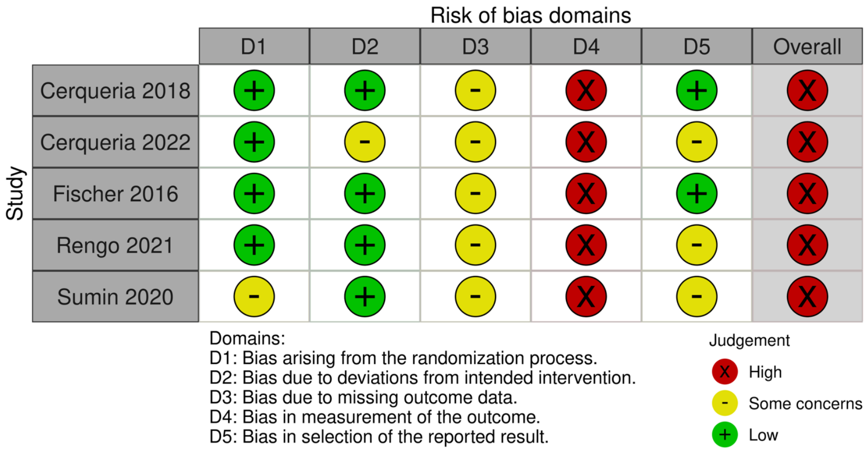
Risk of bias domains
D1
D2
D3
D4
D5
Overall
Study
Cerqueria 2018
Cerqueria 2022
Fischer 2016
Rengo 2021
Sumin 2020
Domains:
D1: Bias arising from the randomization process.
D2: Bias due to deviations from intended intervention.
D3: Bias due to missing outcome data.
D4: Bias in measurement of the outcome.
D5: Bias in selection of the reported result.
Judgement
High
Some concerns
Low
