# Supplementary material for: Efficacy of neuromuscular electrical stimulation for thoracic and abdominal surgery: A systematic review and meta-analysis
Source: PLoS One. 2023 Nov 30;18(11):e0294965. doi: 10.1371/journal.pone.0294965 (PMC10688715; doi:10.1371/journal.pone.0294965)
Supplement: S3 Appendix — Risk of bias summary: Pulmonary surgery (a) Length of stay in ICU (b) Length of stay in hospital. (PDF) [file pone.0294965.s005.pdf]

S5 Appendix: Risk of bias summary: Digestive System surgery (a) Lower limb muscle strength (b) Walking ability (c) Activity of daily living

(a)

Risk of bias domains

Study	D1	D2	D3	D4	D5	Overall
André 2021	+	+	-	+	+	-
Hanada 2019	+	+	-	X	-	X
Hardy 2022	+	-	-	X	X	X

Domains:
D1: Bias arising from the randomization process.
D2: Bias due to deviations from intended intervention.
D3: Bias due to missing outcome data.
D4: Bias in measurement of the outcome.
D5: Bias in selection of the reported result.

Judgement
X High
- Some concerns
+ Low

(b)

Risk of bias domains

Study	D1	D2	D3	D4	D5	Overall
André 2021	+	+	-	+	+	-
Hanada 2019	+	+	-	X	-	X

Domains:
D1: Bias arising from the randomization process.
D2: Bias due to deviations from intended intervention.
D3: Bias due to missing outcome data.
D4: Bias in measurement of the outcome.
D5: Bias in selection of the reported result.

Judgement
X High
- Some concerns
+ Low

(c)

Risk of bias domains

Study	D1	D2	D3	D4	D5	Overall
Hanada 2019	+	+	-	X	-	X

Domains:
D1: Bias arising from the randomization process.
D2: Bias due to deviations from intended intervention.
D3: Bias due to missing outcome data.
D4: Bias in measurement of the outcome.
D5: Bias in selection of the reported result.

Judgement
X High
- Some concerns
+ Low
