# Supplementary material for: Efficacy of neuromuscular electrical stimulation for thoracic and abdominal surgery: A systematic review and meta-analysis
Source: PLoS One. 2023 Nov 30;18(11):e0294965. doi: 10.1371/journal.pone.0294965 (PMC10688715; doi:10.1371/journal.pone.0294965)

S6 Appendix: Risk of bias summary: Pulmonary surgery (a) Length of stay in ICU (b) Length of stay in hospital

(a)

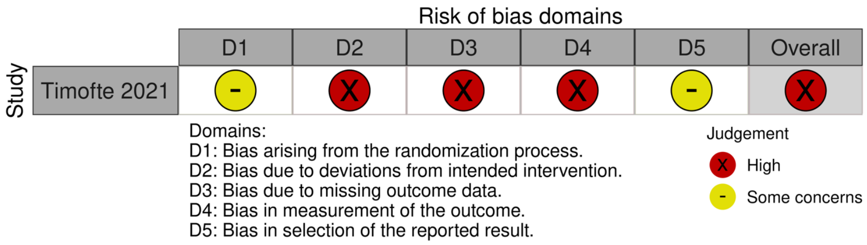

(b)

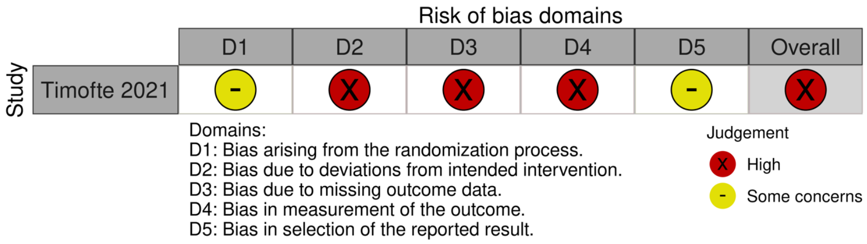

Supplement: S4 Appendix — Risk of bias summary: Digestive system surgery (a) Lower limb muscle strength (b) Walking ability (c) Activity of daily living. (PDF) [file pone.0294965.s006.pdf]
