# Supplementary material for: Efficacy of neuromuscular electrical stimulation for thoracic and abdominal surgery: A systematic review and meta-analysis
Source: PLoS One. 2023 Nov 30;18(11):e0294965. doi: 10.1371/journal.pone.0294965 (PMC10688715; doi:10.1371/journal.pone.0294965)

S7 Appendix: Risk of bias summary: Other surgery (Length of stay in hospital)

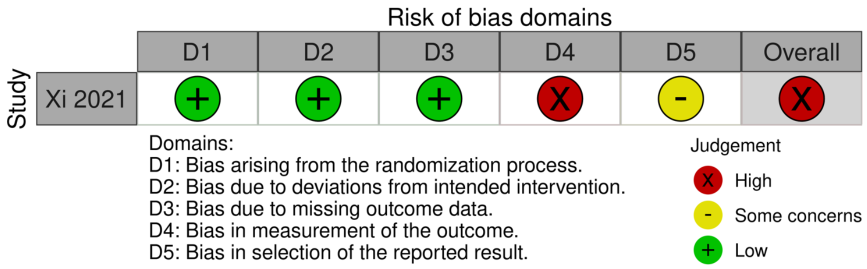

Supplement: S5 Appendix — (PDF) [file pone.0294965.s007.pdf]
