# Supplementary figures and images for: Efficacy of neuromuscular electrical stimulation for thoracic and abdominal surgery: A systematic review and meta-analysis
Source: PLoS One. 2023 Nov 30;18(11):e0294965. doi: 10.1371/journal.pone.0294965 (PMC10688715; doi:10.1371/journal.pone.0294965)

S8 Appendix: Subgroup analysis: Cardiovascular surgery (Lower limb muscle strength)

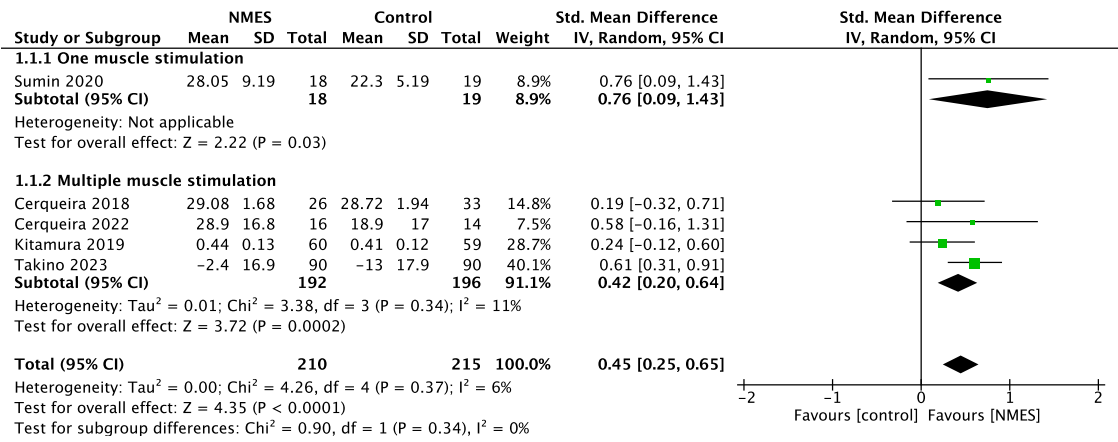

Supplement: S6 Appendix — (PDF) [file pone.0294965.s008.pdf]
